# From Individual Grain Boundaries to Irregular Grain Networks: Drift–Diffusion Simulation of Polycrystalline Silicon Solar Cells

**DOI:** 10.3390/nano16161041

**Published:** 2026-08-21

**Authors:** Irodakhon Gulomova, Oussama Accouche, Zaher Al Barakeh, Rayimjon Aliev, Navruzbek Mirzaalimov, Makhfuza Alinazarova, Jasurbek Gulomov

**Affiliations:** 1 Department of Condensed Matter Physics, Andijan State University, Andijan 170100, Uzbekistan; 2 College of Engineering and Technology, American University of the Middle East, Egaila 54200, Kuwait; oussama.accouche@aum.edu.kw (O.A.);; 3 Department of Physics, Faculty of Physics and Mathematics, Namangan State University, Namangan 160119, Uzbekistan; 4 Andijan State Pedagogical Institute, Andijan 170100, Uzbekistan

**Keywords:** silicon solar cells, grain boundaries, drift–diffusion simulation, polycrystalline silicon, Voronoi tessellation, carrier recombination, device simulation

## Abstract

Grain boundaries (GBs) are important recombination-active defects in polycrystalline and multicrystalline silicon solar cells, but the effects of their electrical activity, geometry, and spatial arrangement are often difficult to separate. In this work, two-dimensional (2D) drift–diffusion simulations are used to investigate how GB trap density, carrier capture cross-section, orientation, length, number, and network geometry affect silicon solar-cell performance. A controlled comparison between rotating GBs whose length changes with angle and fixed-length GBs shows that the strong apparent orientation dependence is dominated by the accompanying variation in active GB length. When the GB length is fixed at 100 μm, the variations in short-circuit current density ($J_{\mathrm{sc}}$), open-circuit voltage ($V_{\mathrm{oc}}$), efficiency, and fill factor are comparatively small. As a second contribution, irregular polycrystalline microstructures are generated by Voronoi tessellation, producing distributions of grain sizes, shapes, boundary lengths, and junctions that are more representative than simplified structures based on isolated or regularly spaced boundaries. These networks are used to connect grain size, total electrically active GB length, recombination, local electric fields, carrier-flow redistribution, and device performance. As the characteristic grain size increases from 5 to 100 μm, $J_{\mathrm{sc}}$ rises from 15 to $34\;\mathrm{mA}\;{\mathrm{cm}}^{-2}$, $V_{\mathrm{oc}}$ from 0.54 to above 0.61 V, and the power conversion efficiency from 6.5% to 17%. GB-induced photovoltaic loss is therefore governed not by GB number or nominal orientation alone, but by the combined effects of electrical activity, total active boundary length, and network geometry.

## 1. Introduction

The increasing global demand for clean and sustainable energy has made photovoltaic technology an important area of scientific research. Solar energy is especially attractive because sunlight is abundant, widely available, and can be converted directly into electricity with low greenhouse-gas emissions [1,2]. As a result, improving the efficiency, stability, and cost-effectiveness of solar cells has become a major goal in the development of future energy technologies [2].

Currently, crystalline-silicon photovoltaic technology includes several important device architectures, such as passivated emitter and rear contact (PERC) solar cells [3], tunnel oxide passivated contact (TOPCon) solar cells [4], silicon heterojunction (SHJ/HJT) solar cells [5], and interdigitated or hybrid back-contact (IBC/HIBC) solar cells [6,7]; flexible silicon-based architectures have also been developed [8]. The theoretical efficiency limit of single-junction crystalline-silicon solar cells is about 29.4%, while the best experimentally demonstrated silicon solar cells have already reached efficiencies above 28% [9,10]. These high efficiencies show the strong development of silicon photovoltaics, but they also indicate that further improvements require a deeper understanding of the remaining loss mechanisms.

Although recent photovoltaic research is increasingly moving toward new material systems, including perovskite solar cells [11], organic solar cells [12], and perovskite/silicon tandem solar cells [13,14], silicon remains the most mature and industrially dominant photovoltaic technology. Because of its technological maturity and wide commercial use, silicon provides an important platform for investigating the physical mechanisms that control solar-cell performance. Continued research on silicon solar cells is therefore necessary to identify the loss processes that limit efficiency and to guide further device optimization.

The efficiency of silicon solar cells is mainly limited by optical losses, resistive losses, and carrier recombination in the bulk material and at device interfaces [15,16,17,18]. In polycrystalline and multicrystalline silicon solar cells, GBs are one important source of recombination because they can contain structural defects and segregated impurities, which reduce the minority-carrier lifetime and diffusion length [19,20,21]. As a result, GBs can decrease $J_{\mathrm{sc}}$, $V_{\mathrm{oc}}$, and fill factor by trapping or recombining photogenerated carriers before they are collected [20,22].

Although many theoretical and experimental studies have investigated the role of GBs in polycrystalline and multicrystalline silicon solar cells, important questions remain regarding how electrical activity and geometry jointly determine device losses [20,23,24,25]. Previous works have shown that GB recombination can reduce carrier lifetime, carrier collection, $V_{\mathrm{oc}}$, fill factor, and efficiency [26,27]. Analytical, TCAD, and experiment-assisted carrier-transport studies have also examined how GB inclination changes recombination and photovoltaic response [26,27,28,29]. However, previous orientation-dependent studies have not always explicitly separated the effect of GB angle from the simultaneous change in active GB length. When a GB is rotated while remaining connected to the borders of a finite device, its total length generally changes with angle. Orientation and active GB length are therefore coupled, so an observed angular dependence cannot automatically be attributed to orientation alone. In addition, many device simulations use average polycrystalline material properties or simplified structures containing isolated straight boundaries, whereas real polycrystalline and multicrystalline silicon contain irregular networks with distributions of grain sizes, shapes, boundary lengths, and junctions [25,27].

Here, these issues are examined in a sequence that progresses from controlled individual GBs to irregular grain networks. First, the effects of trap density and carrier capture cross-section are quantified. Vertical, horizontal, and inclined GBs are then examined, followed by a fixed-length rotation study that removes the confounding variation of GB length and therefore isolates the orientation dependence at constant active length. The effects of GB number and spatial arrangement are also investigated. Finally, irregular 2D polycrystalline microstructures are generated by Voronoi tessellation with random grain shapes and controlled characteristic grain sizes. This approach provides a more realistic representation of GB-network geometry and makes it possible to connect electrical defect activity, total active boundary length, local electrostatic fields, carrier-flow redistribution, recombination, and macroscopic photovoltaic performance.

## 2. Materials and Methods

### 2.1. Device Model

Numerical simulations were carried out with Sesame 2.0, a 2D semiconductor device simulator [30,31,32]. Sesame solves Poisson’s equation together with the electron and hole continuity equations. Carrier currents are calculated from drift in the electric field and diffusion caused by concentration gradients. Generation and recombination enter the continuity equations, and the equations are solved together until a steady state is reached [33]. The simulated device was an n–p silicon solar cell with a total thickness of 175 μm, close to that of a standard wafer-based cell [34,35]. The front n-type emitter was 1.5 μm thick, and the p-type base was 173.5 μm thick. The donor concentration in the emitter was $1\times 10^{17}\;{\mathrm{cm}}^{-3}$, while the acceptor concentration in the base was $1\times 10^{16}\;{\mathrm{cm}}^{-3}$. These values give a simple, well-defined n–p junction while keeping the study focused on GB effects. The lateral width was 100 μm so that several grain sizes and GB arrangements could be compared in the same domain.

The silicon band gap was set to 1.12eV, the electron affinity to 4.05eV, and the relative permittivity to 11.7. The electron and hole mobilities were 1350 and $480\;{\mathrm{cm}}^{2}\;V^{-1}\;s^{-1}$, respectively. These are standard room-temperature values for crystalline silicon. The bulk electron and hole lifetimes were both set to $10^{-4}\;s$, so recombination in the grain interiors was present in every simulation. Radiative and Auger recombination were also included. Trap-assisted recombination in the bulk and at GB defects was described using the Shockley–Read–Hall (SRH) formalism [36,37]. Thus, the calculated response includes bulk loss as a common background, while changes between otherwise identical structures show the added effect of the GBs.

Since this work focused on the recombination effect of GBs rather than detailed optical modeling [38], illumination was approximated by a Beer–Lambert generation profile [39,40],

(1) $$G\left(x\right)=G_{0}\exp\left(-\alpha x\right),$$

where *x* is the depth measured from the illuminated surface, $G_{0}=4.5\times 10^{19}\;{\mathrm{cm}}^{-3}\;s^{-1}$, and $\alpha =120\;{\mathrm{cm}}^{-1}$. The incident power density was set to $100\;\mathrm{mW}\;{\mathrm{cm}}^{-2}$ only for calculating the power conversion efficiency. This single-parameter profile does not represent the wavelength-dependent absorption, reflection, and generation produced by an AM1.5G spectrum [41]. A spectral model would change the depth distribution of generated carriers and therefore the absolute $J_{\mathrm{sc}}$, $V_{\mathrm{oc}}$, and efficiency. It could also change the magnitude of GB losses because boundaries at different depths would overlap differently with the generation profile. The main relative trends are expected to remain, because every GB configuration is compared under the same illumination, but this should be verified in a future optical–electrical calculation.

Ohmic contacts were applied at the front and rear surfaces. The electron and hole surface recombination velocities at the contacts were both set to $10^{7}\;\mathrm{cm}\;s^{-1}$.

The 2D model describes a cross-section that is assumed to continue uniformly in the out-of-plane direction. It is suitable for comparing long GB segments, their angles, and planar grain networks at much lower cost than a 3D calculation. It does not describe finite out-of-plane GB shapes or fully 3D current paths. Therefore, the results are used to compare trends between structures, not to reproduce every feature of a real wafer.

A nonuniform mesh was used in the depth direction. The mesh contained 91 points through the full device thickness. Eighteen mesh points were placed in the 1.5 μm emitter, giving an average spacing of about 0.088 μm in this region. The remaining points were placed in the base, giving an average spacing of about 2.38 μm. In the lateral direction, 41 points were used, corresponding to an average lateral spacing of 2.50 μm.

### 2.2. GB Model

GBs were modeled as recombination-active line defects. Unless a parameter sweep is stated, the reference GB trap density was $2.15\times 10^{11}\;{\mathrm{cm}}^{-2}$. The reference electron and hole capture cross-sections were both $10^{-15}\;{\mathrm{cm}}^{2}$, and the defect energy was placed at the intrinsic Fermi level, where an SRH center has a strong recombination effect. These values are within the ranges commonly used to describe active GB defects in silicon models, although experimental values vary with GB character, impurities, and passivation [25,42,43]. They provide a moderately active reference boundary; the trap-density and capture-cross-section sweeps test how sensitive the conclusions are to this choice.

To study the effects of GB properties and geometry, several parameters were varied systematically. These included trap density, carrier capture cross-section, GB position, orientation, spacing between boundaries, number of boundaries, and grain-network geometry. “Vertical” GBs extend mainly through the device depth and are approximately normal to the illuminated surface. “Horizontal” GBs extend mainly across the device width and are approximately parallel to that surface. Irregular polycrystalline structures were generated using reproducible Voronoi tessellations [44]. In the 2D model, GBs are line features in the simulated cross-section and represent planar defects that continue out of the plane. The spatially integrated recombination quantity reported below is defined as

(2) $$R_{\mathrm{tot}}=\int_{A}R\left(x,y\right)\;dA,$$

which has units of ${\mathrm{cm}}^{-1}\;s^{-1}$ in the 2D formulation and corresponds to recombination per unit out-of-plane depth.

The Voronoi study covers characteristic grain sizes from 5 to 100 μm. Within the present drift–diffusion model, changing grain size changes the total active GB length and the positions of recombination and electrostatic barriers. The model does not include a separate ballistic or grain-boundary-reflection term. It also does not describe thermodynamic size effects that can change the material properties of very small grains. Consequently, the grain-size results isolate the effects represented by the continuum model and should not be extended to nanoscale grains without an additional transport model.

## 3. Results and Discussion

### 3.1. Effects of Capture Cross-Section and Trap Density

**Figure 1:**
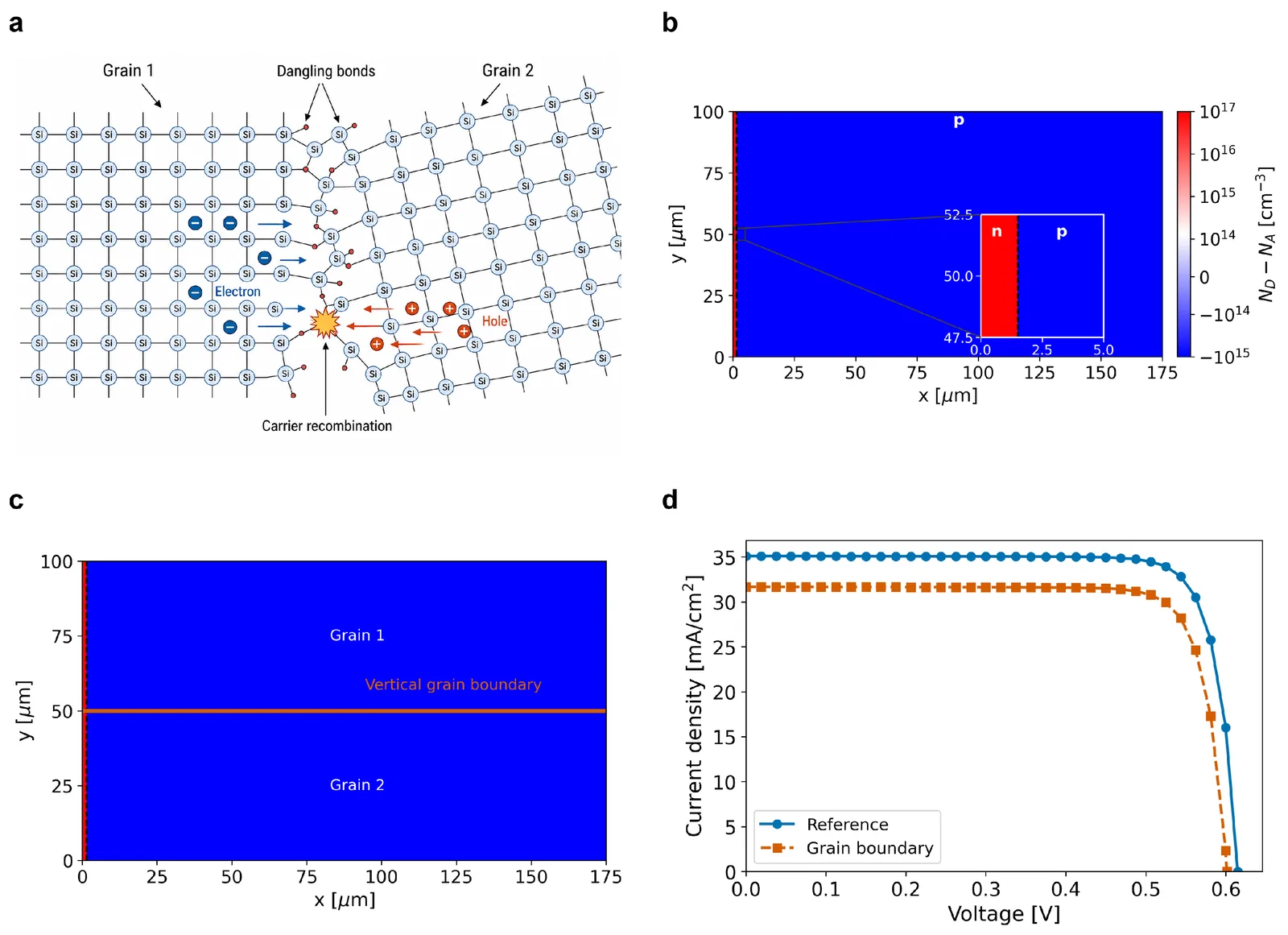
Schematic illustration of the GB and the simulated silicon solar cells. (**a**) Formation of a GB between two silicon grains with different crystal orientations. (**b**) Reference silicon solar cell without a GB, where n denotes the n-type emitter and p denotes the p-type base. The color scale shows the prescribed net doping concentration, not the free-carrier concentration; the abrupt color change represents the step-like doping profile. (**c**) Silicon solar cell containing a GB between Grain 1 and Grain 2. (**d**) I–V characteristics of the reference solar cell and the solar cell with a GB.

GBs are formed when crystal domains with different orientations meet. At these boundaries, the periodic atomic structure is disturbed, and dangling bonds or impurity-related defects can appear (see Figure 1a). These defects introduce energy levels inside the band gap and act as Shockley–Read–Hall (SRH) recombination centers [33,36,37]. Such boundaries can reduce carrier lifetime and degrade the photovoltaic performance of silicon solar cells [42,45,46].

The reference solar cell structure used in this work is shown in Figure 1b. To study the influence of GB recombination, a vertical GB was introduced into the absorber (see Figure 1c). The comparison of the current–voltage curves shows that the GB reduces both $J_{\mathrm{sc}}$ and $V_{\mathrm{oc}}$ (see Figure 1d). This decrease occurs because photogenerated electrons and holes can recombine at the defect states before being collected. In addition, enhanced recombination increases the dark saturation current, which lowers the quasi-Fermi-level splitting and therefore reduces $V_{\mathrm{oc}}$ [33,47].

The electrical activity of a GB depends strongly on the trap density ($N_{t}$) and the carrier capture cross-section (σ). In the SRH model, the recombination strength of a planar defect is related to the surface recombination velocity,

(3) $$S_{n,p}=\sigma_{n,p}v_{\mathrm{th}}N_{t},$$

where $v_{\mathrm{th}}$ is the thermal velocity. This relation shows that recombination increases when either the number of trap states or their ability to capture carriers increases. The same capture cross-section was used in all simulations for electrons and holes, i.e., $\sigma_{n}=\sigma_{p}=\sigma$.

**Figure 2:**
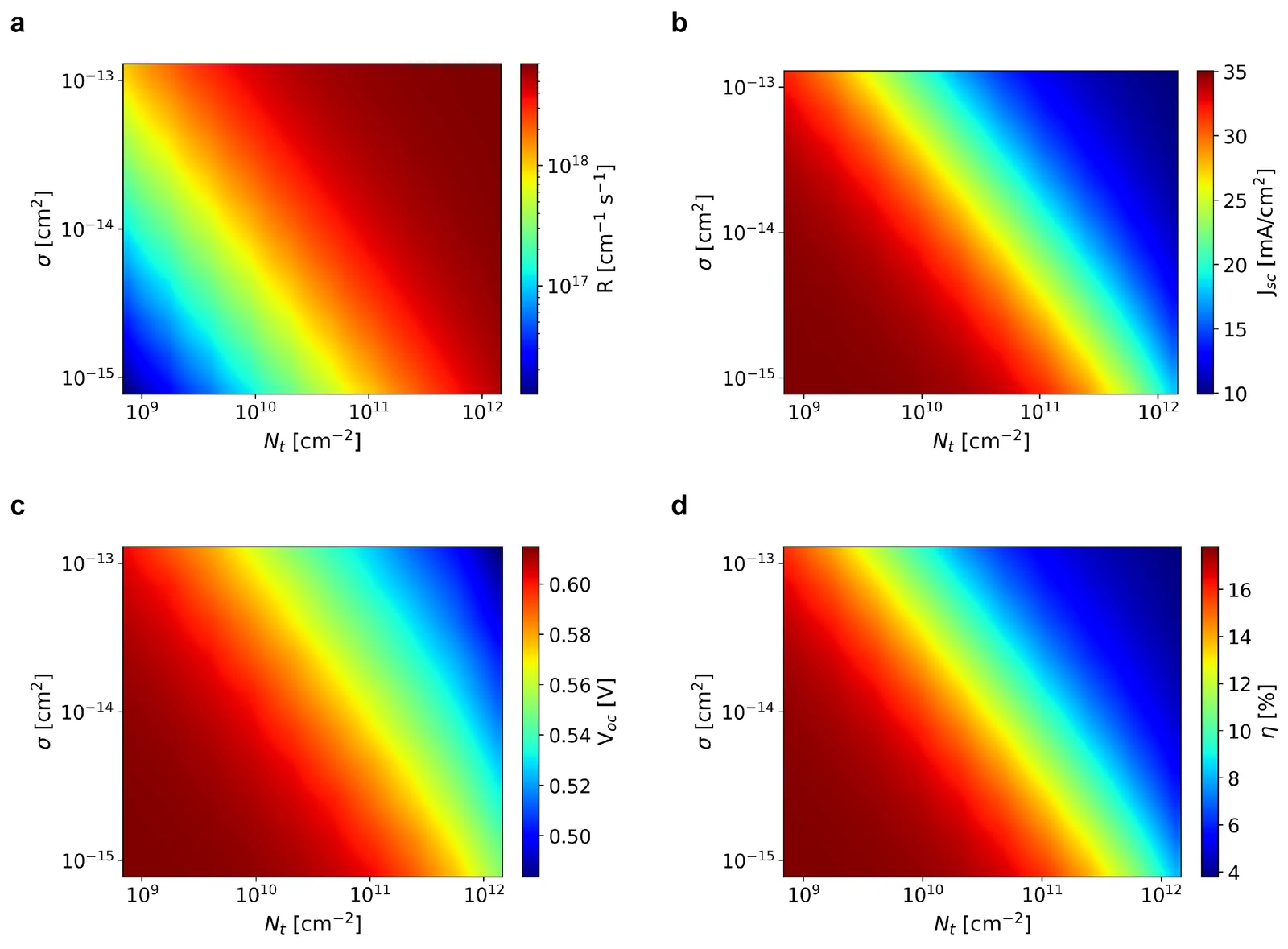
The dependence of total recombination rate (**a**), $J_{\mathrm{sc}}$ (**b**), $V_{\mathrm{oc}}$ (**c**), and efficiency (**d**) of a silicon solar cell with a vertical GB on carrier capture cross-section and GB trap density.

The dependence of the device parameters on $N_{t}$ and σ is presented in Figure 2 and Figure S1. The total recombination rate increases as both parameters increase (see Figure 2a). The diagonal trend in the map indicates that the recombination activity depends jointly on σ and $N_{t}$. A GB can therefore become highly recombination-active either because it contains many traps or because the existing traps have a large capture cross-section.

As a direct result of stronger recombination, $J_{\mathrm{sc}}$, $V_{\mathrm{oc}}$, and the efficiency decrease with increasing $N_{t}$ and σ (see Figure 2b–d). The reduction in $J_{\mathrm{sc}}$ is caused by the loss of photogenerated carriers at the GB before collection. The decrease in $V_{\mathrm{oc}}$ follows from the increase in recombination current and dark saturation current. Since

(4) $$V_{\mathrm{oc}}\approx \frac{k_{B}T}{q}\ln\left(\frac{J_{\mathrm{sc}}}{J_{0}}+1\right),$$

a higher $J_{0}$ leads to a lower $V_{\mathrm{oc}}$ [47]. Consequently, the efficiency follows the same decreasing trend.

The trends confirm that photovoltaic performance depends strongly on GB electrical quality. Reducing either the trap density or the capture cross-section can suppress GB recombination and improve device performance. They also underscore the importance of GB passivation in silicon solar cells [22,42].

### 3.2. Separating GB Orientation from Active Boundary Length

GB orientation can affect recombination because it changes the geometrical relation between the boundary, the junction, and the carrier-flow direction [26,27,28]. Experiments have also shown that GB inclination should be considered when interpreting local recombination activity [29]. However, orientation and GB length can change at the same time in a finite simulation domain. The main question here is therefore whether the observed angular dependence is caused by orientation itself or mainly by the accompanying change in active GB length. Numerical angles should also be compared carefully across different studies because the angular definition, device dimensions, and GB constraints may not be the same.

**Figure 3:**
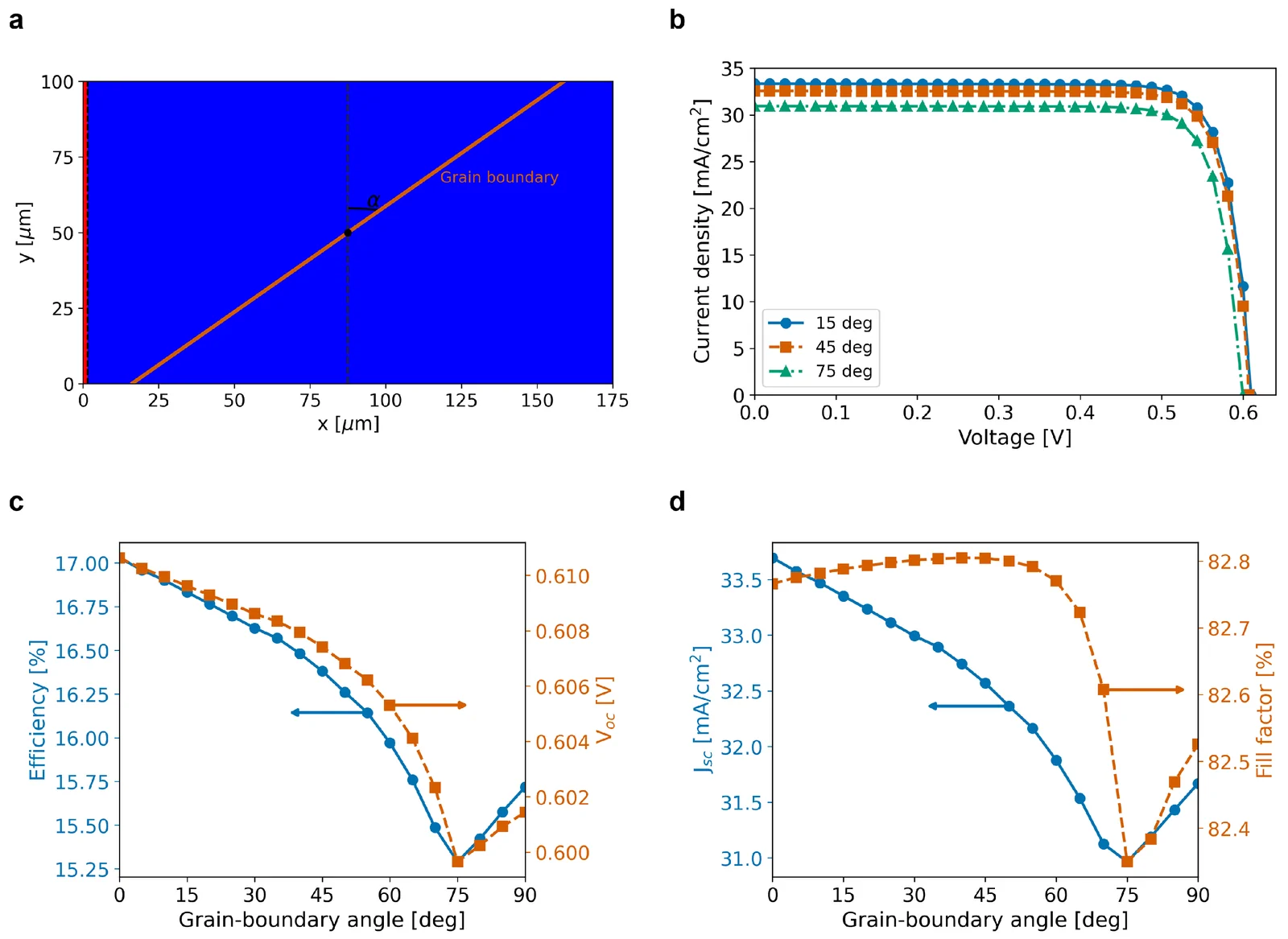
Effect of GB orientation on the performance of the silicon solar cell when the GB length changes with angle. (**a**) Schematic of the silicon solar cell containing an inclined GB. The orange line shows the GB, which is rotated by an angle α; the dashed lines mark the n–p junction and the vertical reference direction used to define α. (**b**) Current–voltage characteristics for GB angles of 15°, 45°, and 75°. (**c**) Dependence of efficiency and $V_{\mathrm{oc}}$ on GB angle. (**d**) Dependence of $J_{\mathrm{sc}}$ and fill factor on GB angle. In (**c**,**d**), the colored arrows associate each curve with the correspondingly colored vertical axis.

To separate these effects, two configurations were studied. In the first, the GB remained connected to two borders of the solar cell while it was rotated (Figure 3a). Its total length therefore changed with angle. In the second, the GB length was fixed at 100 μm and only its orientation was changed by rotating it about the center of the device. A longer GB has a greater recombination-active boundary length and can interact with a larger fraction of the photogenerated carriers.

In the border-connected configuration, the current–voltage characteristics change strongly with GB angle (Figure 3b). As the angle increases from 0° to 75°, the efficiency decreases from 17.0% to 15.3% and then partly recovers at 90° (Figure 3c). Over the same range, $V_{\mathrm{oc}}$ decreases from 0.610 to 0.600 V, while $J_{\mathrm{sc}}$ decreases from 33.7 to $31.0\;\mathrm{mA}\;{\mathrm{cm}}^{-2}$ (Figure 3d). Thus, $J_{\mathrm{sc}}$, $V_{\mathrm{oc}}$, and efficiency reach their lowest values near 75°.

The fill factor shows a smaller and slightly different trend. It increases weakly from 0° to 45° and then decreases toward 75°. This small initial increase does not indicate an overall improvement because both $J_{\mathrm{sc}}$ and $V_{\mathrm{oc}}$ decrease over the same angular range. It mainly reflects a small change in the shape of the current–voltage curve and in the position of the maximum-power point.

The strongest performance loss near 75° occurs at almost the same angle as the maximum integrated recombination rate shown in Figure S2. This shows that the angular variation in device performance is directly linked to stronger GB recombination. Previous studies have also predicted orientation-dependent GB recombination, including cases where inclination changes the effective length of the recombination-active region [26,27,28]. For the border-connected geometry used here, however, the physical GB length also changes during rotation. The strong angular dependence cannot be attributed to orientation alone. In particular, the minimum near 75° is not a universal critical angle. Its position depends on the device dimensions, aspect ratio, angular definition, and the requirement that the GB connect two borders of a finite domain.

**Figure 4:**
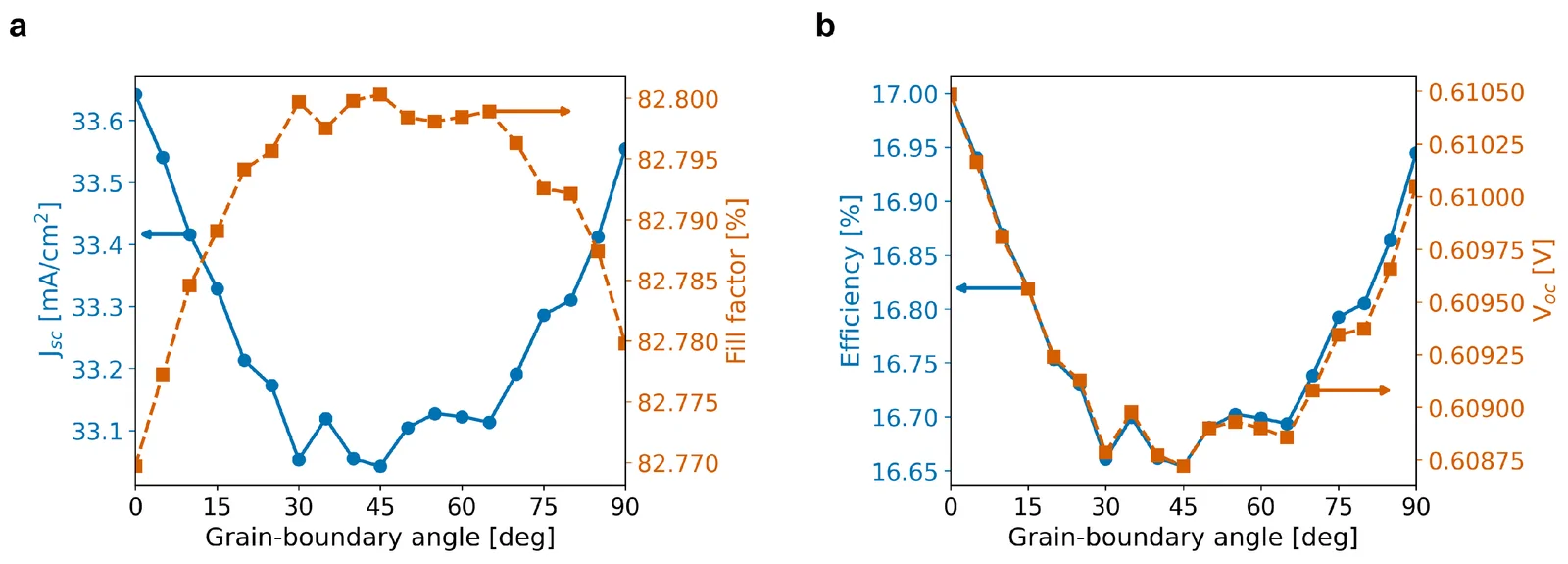
Effect of GB orientation on the photovoltaic parameters when the GB length is fixed at 100 μm. (**a**) Dependence of $J_{\mathrm{sc}}$ and fill factor on GB angle. (**b**) Dependence of efficiency and $V_{\mathrm{oc}}$ on GB angle. The colored arrows associate each curve with the correspondingly colored vertical axis.

To remove the simultaneous change in active GB length, the simulations were repeated with *L*_GB_ = 100 μm for all angles. The fixed-length geometry is shown in Figure S3, and the photovoltaic parameters are shown in Figure 4. In this case, the angular dependence becomes much weaker. The efficiency, $V_{\mathrm{oc}}$, and $J_{\mathrm{sc}}$ show shallow minima near 45°, while the fill factor shows a small maximum.

The difference between the two configurations is clear. In the border-connected case, the efficiency changes by 1.7 percentage points, $J_{\mathrm{sc}}$ by $2.7\;\mathrm{mA}\;{\mathrm{cm}}^{-2}$, and $V_{\mathrm{oc}}$ by 10 mV between the best and worst orientations. At fixed length, the corresponding changes are only 0.35 percentage points in efficiency, $0.6\;\mathrm{mA}\;{\mathrm{cm}}^{-2}$ in $J_{\mathrm{sc}}$, and less than 2 mV in $V_{\mathrm{oc}}$. The fill-factor variation is only 0.03 percentage points. Thus, fixing the GB length greatly reduces the apparent orientation dependence under the present device and defect conditions.

The weaker fixed-length response does not imply that GB orientation has no electrical effect. Earlier theoretical and TCAD studies show that orientation can change recombination by changing how the boundary intersects the carrier-flow direction and carrier-concentration field [26,28]. In the fixed-length study, the GB is rotated about the center of the device and does not intersect the shallow n–p junction. Rotation nevertheless changes the depth and lateral positions of its two ends and its overlap with the nonuniform generation and carrier-flux fields. Near 45°, the boundary has comparable depth and lateral projections, giving a slightly larger overlap with these transport paths in this finite geometry. This provides a plausible origin of the shallow minimum. Because the changes are small, the feature is considered geometry-specific and not a universal critical orientation.

### 3.3. Effect of GB Number and Spatial Arrangement

Previous 2D studies have shown that GB-induced losses depend on several factors, including grain size, GB recombination velocity, potential barriers, and boundary geometry [24,48]. Nevertheless, the number of GBs alone is not sufficient to predict device performance. Two structures with the same number of GBs can have different total boundary lengths, positions, orientations, spacings, and relations to carrier-transport pathways.

The vertical and horizontal configurations have different active lengths in the finite simulation domain. Their comparison therefore contains both orientation and length effects.

**Figure 5:**
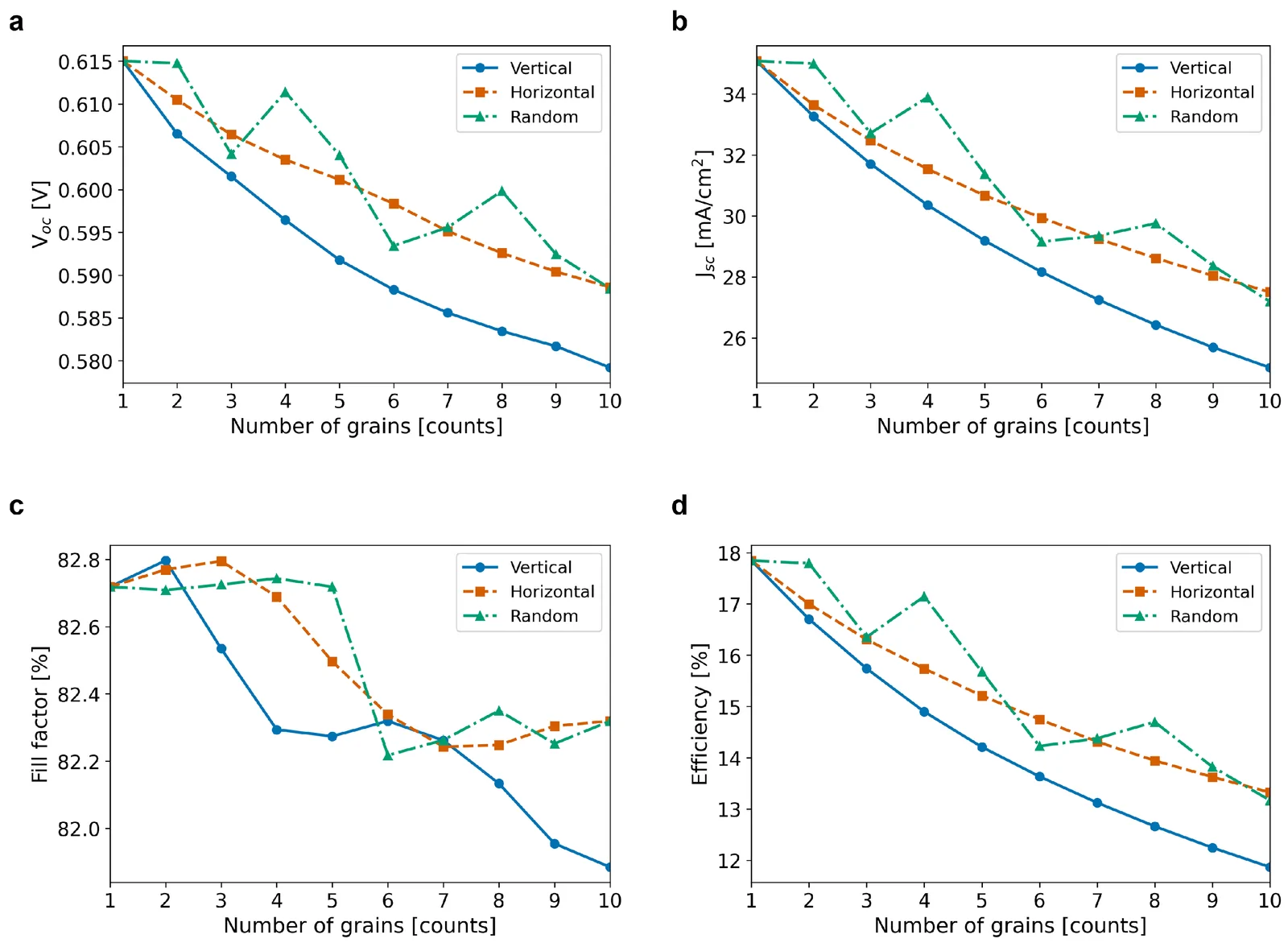
Effect of GB number on the photovoltaic parameters of the silicon solar cell for vertical, horizontal, and random GB configurations: (**a**) $V_{\mathrm{oc}}$, (**b**) $J_{\mathrm{sc}}$, (**c**) fill factor, and (**d**) power conversion efficiency.

As shown in Figure 5, the photovoltaic parameters generally decrease as the number of active GBs increases. The strongest degradation occurs for the vertical configuration. When the number of vertical GBs increases from 1 to 10, $V_{\mathrm{oc}}$ decreases from 0.615 to 0.579 V, $J_{\mathrm{sc}}$ from 35.0 to $25.0\;\mathrm{mA}\;{\mathrm{cm}}^{-2}$, and the efficiency from 17.8% to 11.9% (Figure 5a,b,d). In this geometry, these boundaries extend through a larger part of the absorber and provide a greater total recombination-active boundary length. Increasing their number also reduces the average spacing between neighboring active GBs. This makes it more likely that photogenerated minority carriers encounter a recombination-active boundary before collection [26,48,49].

The horizontal boundaries show weaker degradation. The random configuration also shows an overall decrease, but with local fluctuations. Representative current–voltage curves for five vertical, five horizontal, and five randomly arranged GBs are compared in Figure S4. The fluctuations in the random series occur because changing the number of random GBs also changes their lengths, positions, and orientations. Adding one boundary therefore does not always produce a proportional increase in the effective recombination loss.

The distinction between GB number and GB activity is even more important in real multicrystalline silicon. Experiments have shown that different GBs can have very different recombination activity because of differences in crystallographic character, impurity segregation, thermal processing, and passivation or gettering history [20,50,51,52]. All simulated GBs have the same trap parameters. This removes boundary-to-boundary electrical differences and allows the geometrical effect to be studied more clearly. Even under this controlled assumption, GB number alone does not determine photovoltaic loss.

The main message of Figure 5 is therefore not simply that more GBs cause more loss. GB count is an incomplete measure. Total active GB length or GB line density gives a better measure of the amount of recombination-active boundary, while the remaining differences depend on GB position and arrangement relative to carrier generation and transport.

### 3.4. Grain-Network Geometry, Recombination, and Device Performance

The previous sections considered individual or simplified GB arrangements. Real polycrystalline silicon contains irregular networks of grains with different sizes, shapes, boundary lengths, and junctions. To introduce this geometrical disorder in a controlled way, 2D synthetic polycrystalline structures were generated by Voronoi tessellation. The aim is not to reproduce a specific experimental wafer. Instead, the Voronoi structures are used to connect grain size, total active GB length, recombination, and device performance. Most previous drift–diffusion studies of polycrystalline silicon solar cells have used simplified, columnar, or isolated GB geometries [24,25,26,53]. The model extends these studies by directly modeling irregular 2D Voronoi networks as electrically active line defects.

**Figure 6:**
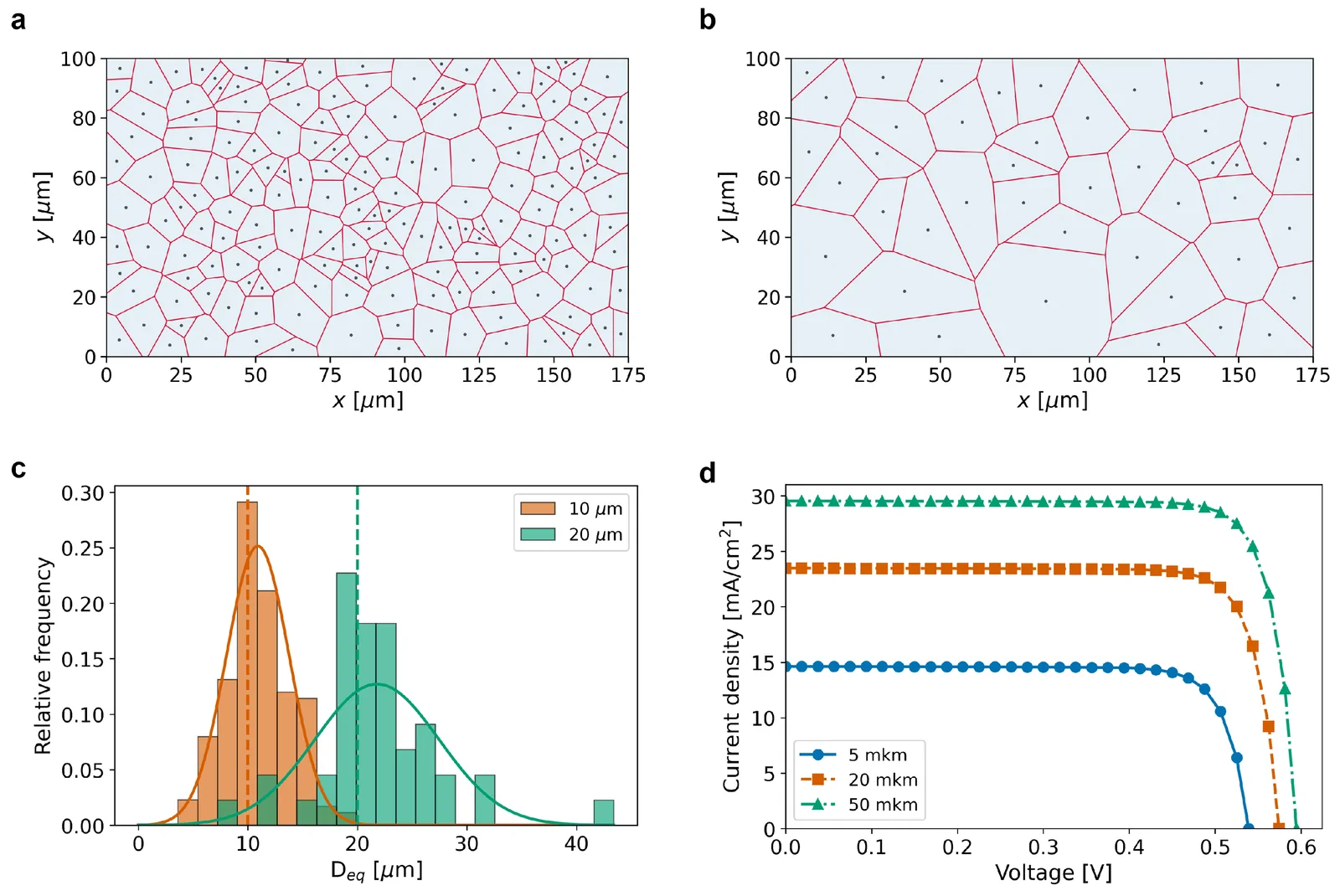
Synthetic irregular polycrystalline silicon structures generated by Voronoi tessellation. (**a**,**b**) Simulated grain structures with target average equivalent grain diameters of 10 μm (**a**) and 20 μm (**b**). The points indicate the Voronoi seeds and the red lines show the GBs. (**c**) Distribution of equivalent grain diameters for structures with target average grain sizes of 10 and 20 μm. The dashed vertical lines indicate the corresponding target values. (**d**) Current–voltage characteristics of silicon solar cells with average grain sizes of 5, 20, and 50 μm.

Because the seed positions are random, the grains are not identical in size. Each structure contains a distribution of equivalent grain diameters around the selected target value (Figure 6c). Increasing the target grain size shifts the distribution toward larger diameters and gives a broader absolute spread. This type of size variation is qualitatively consistent with experimental polycrystalline and multicrystalline silicon microstructures [54,55,56]. The Voronoi model therefore gives a more representative geometry than equally spaced straight boundaries, although it remains a synthetic 2D structure rather than an experimentally reconstructed wafer.

**Figure 7:**
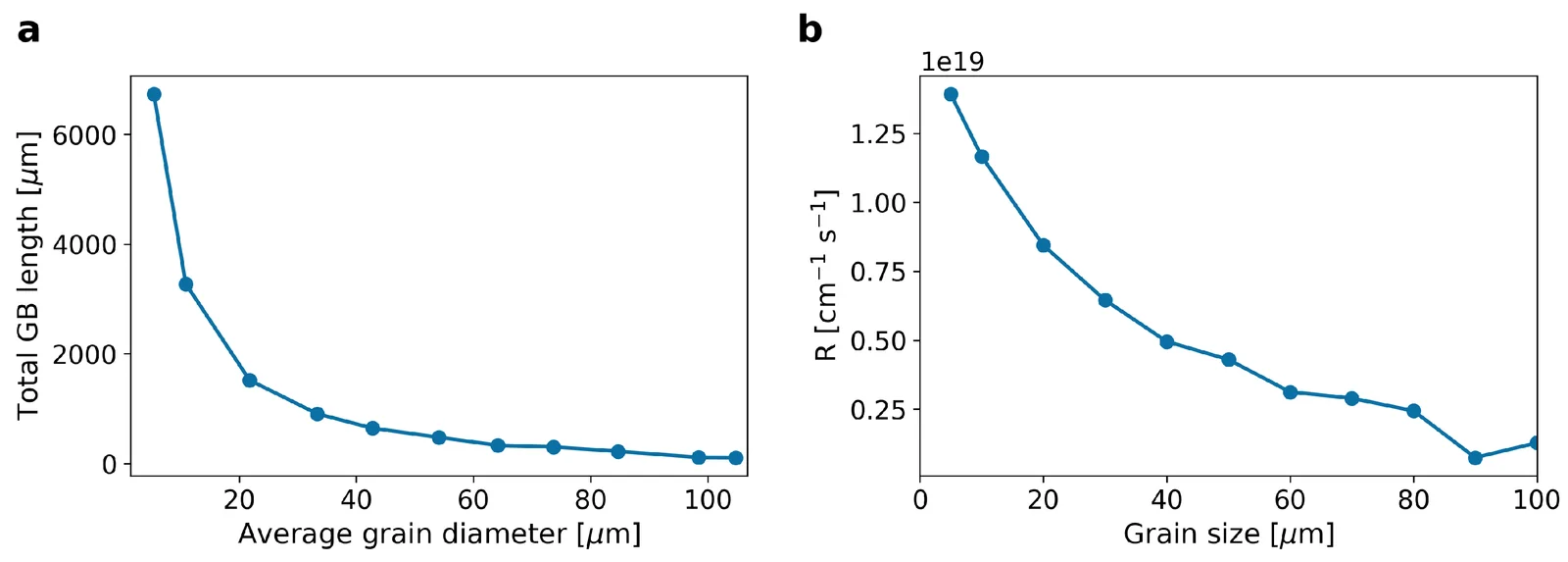
Effect of average grain size on GB geometry and recombination. (**a**) Total GB length as a function of average grain diameter. (**b**) Spatially integrated total recombination rate as a function of average grain size.

The connection between grain size and recombination begins with the amount of active GB. For a fixed simulated area, smaller grains lead to more grains and a larger total GB length. In a simple scaling picture, the number of grains varies approximately as $A/D_{g}^{2}$, while the perimeter of one grain scales as $D_{g}$. Therefore,

(5) $$L_{\mathrm{GB}}\propto \frac{A}{D_{g}},$$

where *A* is the simulated cross-sectional area and $D_{g}$ is the characteristic grain diameter. This is an approximate relation, not an exact law. Finite-domain effects, shared edges, boundary truncation at the simulation perimeter, and the random Voronoi geometry all cause deviations. Even so, Figure 7a shows the expected overall trend: the total GB length decreases rapidly as the average grain size increases.

All GBs in the model have the same defect density, capture cross-sections, and defect energy. Reducing the total GB length therefore reduces the total amount of recombination-active boundary. As a result, the integrated recombination rate decreases strongly as the grain size increases (Figure 7b). This trend agrees with earlier theory, simulations, and spatially resolved experiments showing that electrically active GBs can reduce minority-carrier lifetime and carrier collection through recombination [20,24,42,48,57]. This work extends these studies by modeling an irregular GB network and directly connecting grain size with total GB length and integrated recombination.

**Figure 8:**
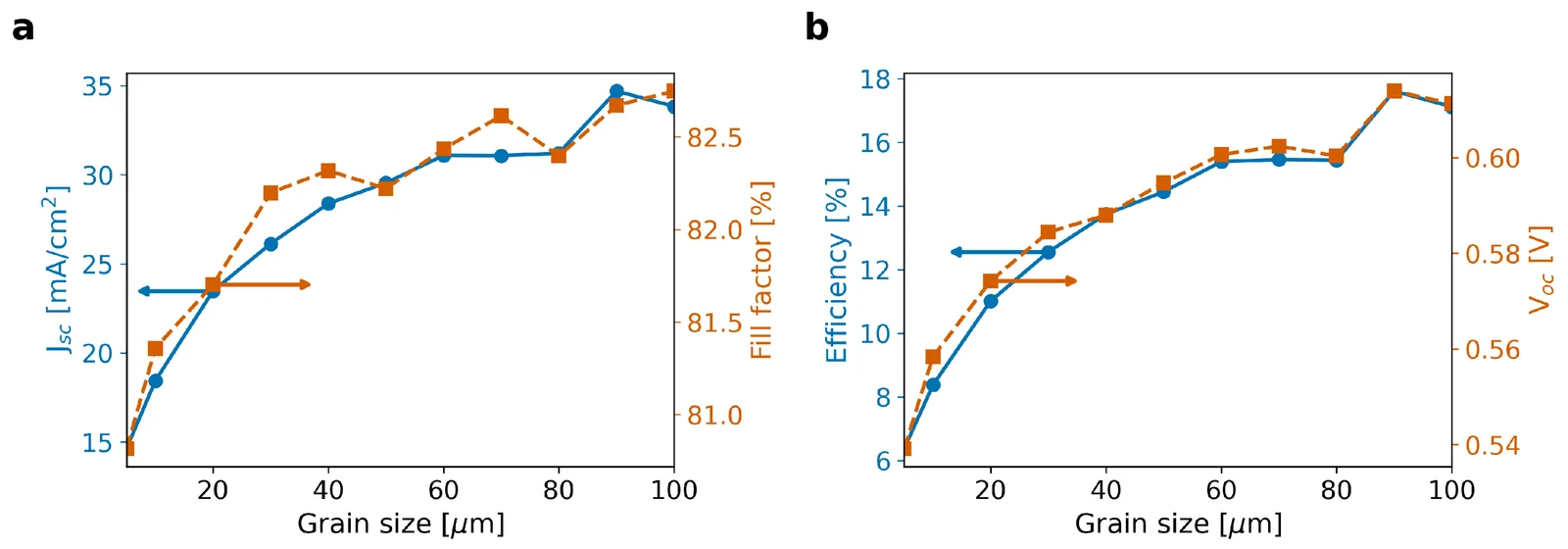
Dependence of the photovoltaic parameters on the average grain size of the synthetic irregular Voronoi polycrystalline silicon structures. (**a**) $J_{\mathrm{sc}}$ (left *y*-axis) and fill factor (right *y*-axis). (**b**) Power conversion efficiency (left *y*-axis) and $V_{\mathrm{oc}}$ (right *y*-axis).

The current–voltage characteristics in Figure 6d show the corresponding improvement in device performance. For the smallest average grain size of 5 μm, $J_{\mathrm{sc}}$ is $15\;\mathrm{mA}\;{\mathrm{cm}}^{-2}$. It rises to $30\;\mathrm{mA}\;{\mathrm{cm}}^{-2}$ at 50 μm. The complete photovoltaic trends are shown in Figure 8.

As the characteristic grain size increases from 5 to 100 μm, $J_{\mathrm{sc}}$ increases from 15 to $34\;\mathrm{mA}\;{\mathrm{cm}}^{-2}$, $V_{\mathrm{oc}}$ from 0.54 to above 0.61 V, and the efficiency from 6.5% to 17%. In comparison, the fill factor changes by only 2%. These different changes suggest that the main GB-related losses in the present model come from recombination and carrier collection rather than from a large change in the squareness of the current–voltage curve.

The reduction in recombination explains the improvement in the photovoltaic parameters. In small-grain networks, the larger active GB length gives photogenerated minority carriers more opportunities to recombine before collection, which lowers $J_{\mathrm{sc}}$. Stronger recombination also increases the recombination current and reduces the electron–hole quasi-Fermi-level splitting under illumination. This lowers $V_{\mathrm{oc}}$ [43,47]. The recovery of both $J_{\mathrm{sc}}$ and $V_{\mathrm{oc}}$ at larger grain size is therefore consistent with the decrease in integrated recombination shown in Figure 7b.

The calculated grain-size trend agrees qualitatively with earlier models in which larger grains reduce the influence of GBs and improve carrier lifetime or photovoltaic performance [48,57]. However, direct quantitative comparison is difficult because previous studies use different device structures, absorber thicknesses, generation profiles, bulk lifetimes, GB parameters, and network geometries. For example, earlier 2D simulations of a particular poly-Si thin-film cell found especially strong sensitivity of $V_{\mathrm{oc}}$ to GB recombination [24]. For the dense irregular networks considered here, small grain size also causes a large $J_{\mathrm{sc}}$ loss. These results are not contradictory. They show that the relative current and voltage losses depend on the device and microstructure being modeled.

The simulated grain-size trend does not imply that larger grains always produce better real multicrystalline-silicon solar cells. Our simulations isolate the effect of GB geometry by assigning the same electrical properties to every boundary. Real multicrystalline silicon is more complex because different GBs can have different crystallographic structures, impurity concentrations, passivation states, and relations to dislocation formation [20,50,56]. Some boundaries can be weakly active or well passivated, while others remain strong recombination centers. Accordingly, the results represent a controlled study of GB geometry in a uniformly active network, not a complete reconstruction of a specific wafer.

At the largest target grain sizes, the grain dimension becomes comparable with the finite simulation domain. Only a few grains and GBs then remain, so one long or favorably placed boundary can noticeably change the result. One reproducible random structure was used for each grain size. The spikes and small non-monotonic changes in Figure 7b and Figure 8 therefore come mainly from differences in the total boundary length, boundary position, and network junctions of these individual structures. They are not interpreted as new physical size transitions or as bulk ensemble averages.

**Figure 9:**
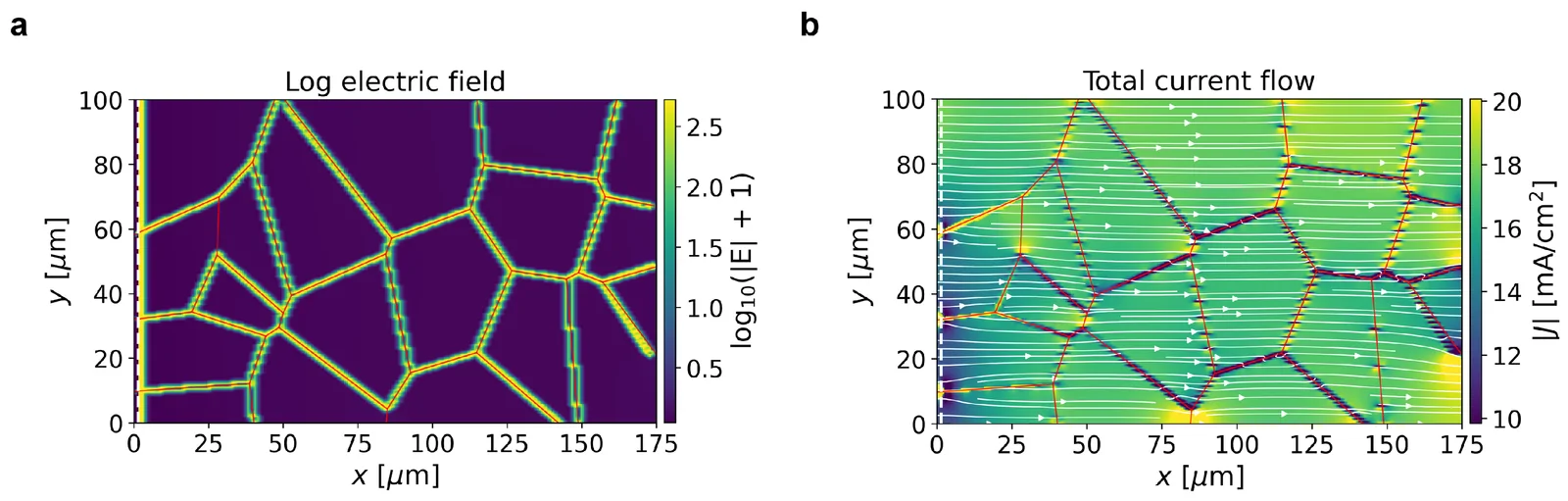
Spatial distributions of the electric field and total current flow in a silicon solar cell containing a synthetic irregular Voronoi GB network with an average grain size of 20 μm. (**a**) Logarithmic magnitude of the electric field, showing strong field localization along electrically active GBs. (**b**) Magnitude and streamlines of the total current density, showing local redistribution of carrier flow around the GB network. The red lines trace the synthetic GB network in both panels.

GBs can also affect carrier transport through local electrostatic effects. Charged GB states can cause band bending and potential barriers that change carrier concentrations and transport [42,43,58]. Sesame includes this effect through trap charge in Poisson’s equation and the resulting drift–diffusion current. However, it does not include an independent reflection probability of the type used in ballistic, Sharvin, or grain-boundary-scattering resistance models. Such a term is more important when the carrier mean free path is comparable to the grain size. Here, the 5–100 μm grains are treated with a continuum drift–diffusion model, and reflection without trapping is outside the scope of the study. Experiments have measured potential barriers at individual GBs in polycrystalline silicon and shown that their magnitude depends on boundary character and impurity contamination [59]. Figure 9a shows strong electric-field localization along the irregular GB network, while the grain interiors generally have much weaker fields. This is consistent with a local electrostatic disturbance near active boundaries, although the field map alone cannot separate trapped-charge effects from other spatial changes in the device.

The current-flow map in Figure 9b shows another local effect of the GB network. The current streamlines are distorted near GBs and especially near boundary junctions. Additional results for a single highly active GB and separate electron- and hole-current maps for the irregular network are shown in Figures S5 and S6. These maps directly show that the GB network redistributes local carrier flow in the simulation. Models of charged GBs further show that recombination and electrostatic barriers can be coupled, so an active boundary may affect both carrier lifetime and local transport [43,58]. The strength of these effects depends on trap occupation, defect density, capture cross-sections, doping, local carrier concentration, and the position of the GB network relative to the main transport pathways.

The main result of the Voronoi analysis can be summarized by the sequence

$$D_{g}↑\;⟹\;L_{\mathrm{GB}}↓\;⟹\;R_{\mathrm{tot}}↓\;⟹\;J_{\mathrm{sc}}↑,\;V_{\mathrm{oc}}↑,\;\eta ↑.$$

Under the controlled assumption that all GBs have the same electrical activity, and larger grains reduce the total active GB length, lower recombination, and improve device performance. The spatial maps also show that the irregular GB network can change local electric fields and carrier-flow paths in addition to acting as a recombination-active defect network.

### 3.5. Relation to Experimental Measurements

The GB parameters can be expressed as a recombination velocity. For electrons or holes,

(6) $$S_{\mathrm{GB}}=\sigma v_{\mathrm{th}}N_{t}.$$

For the reference values and a thermal velocity of order $10^{7}\;\mathrm{cm}\;s^{-1}$, $S_{\mathrm{GB}}$ is of order $2\times 10^{3}\;\mathrm{cm}\;s^{-1}$. This quantity can be compared more directly with GB recombination velocities inferred from spatially resolved lifetime, photoluminescence, or electron-beam-induced-current measurements [22,49,50]. It is an effective SRH value, not a universal property of every GB.

An experimental effective lifetime contains several losses. In a simple additive approximation,

(7) $$\frac{1}{\tau_{\mathrm{eff}}}\approx \frac{1}{\tau_{\mathrm{bulk}}}+S_{\mathrm{GB}}\frac{A_{\mathrm{GB}}}{V}+\frac{2S_{\mathrm{surf}}}{W},$$

where $A_{\mathrm{GB}}/V$ is the active GB area per sample volume, $S_{\mathrm{surf}}$ is the surface recombination velocity, and *W* is the sample thickness. This expression is approximate because injection level, GB charge, and carrier diffusion can make the contributions nonuniform. Experimentally, bulk and surface losses can be estimated from well-passivated reference samples and thickness-dependent measurements. Spatially resolved lifetime or luminescence maps can then be compared with the GB positions found by optical imaging or electron backscatter diffraction. The remaining local lifetime reduction gives an estimate of the GB contribution [20,22].

The Voronoi networks reproduce several geometrical features of real material, including distributions of grain size, boundary length, angle, and junctions. They do not reproduce measured crystallographic boundary types, impurity segregation, dislocations, or the fact that different GBs have different electrical activity [50,56]. They are therefore controlled synthetic networks rather than reconstructions of a particular wafer. For experiments, the simulations suggest two complementary routes for reducing loss: reduce the amount of active GB area by increasing grain size, and reduce $S_{\mathrm{GB}}$ by passivation. Passivation is especially important because a large but electrically inactive boundary causes little SRH loss, whereas a smaller highly active boundary can still be harmful [21].

### 3.6. Main Limitations

This study uses a single-coefficient optical generation profile rather than a wavelength-resolved AM1.5G model. It also uses one random Voronoi realization for each target grain size, so small changes between neighboring points contain statistical variation from the particular network. In addition, the 2D geometry and uniform electrical activity assigned to all GBs simplify the three-dimensional and boundary-dependent behavior of real material. The results should therefore be read as controlled trends under common assumptions rather than exact predictions for a specific wafer.

## 4. Conclusions

This work used 2D drift–diffusion simulations to determine how the electrical activity and geometry of grain boundaries jointly affect polycrystalline silicon solar-cell performance. The analysis progressed from controlled individual boundaries to multiple-boundary configurations and irregular Voronoi networks, allowing trap density, capture cross-section, active boundary length, orientation, number, spatial arrangement, and characteristic grain size to be interpreted within one device framework.

The controlled orientation comparison provides a particularly important distinction. When a border-connected GB is rotated, its active length changes simultaneously with angle, and the photovoltaic parameters show a pronounced angular dependence. When the GB length is fixed at 100 μm, the variations in $J_{\mathrm{sc}}$, $V_{\mathrm{oc}}$, efficiency, and fill factor become much smaller. Thus, under the modeled conditions, most of the strong apparent orientation dependence originates from the accompanying change in active GB length, while the residual fixed-length orientation effect is secondary. This result shows that orientation and active length should be separated whenever the physical origin of an angular performance trend is interpreted.

The multiple-boundary simulations further show that GB count is not a sufficient descriptor of photovoltaic loss. Configurations with the same number of boundaries can differ in total active length, spacing, orientation, and spatial overlap with carrier-transport pathways. A second important contribution is the direct drift–diffusion modeling of irregular Voronoi grain networks containing distributions of grain sizes, shapes, boundary lengths, and junctions. These networks provide a more representative description of polycrystalline geometry than isolated or regularly spaced straight boundaries. Increasing the characteristic grain size from 5 to 100 μm decreases the total GB length and integrated recombination rate and produces substantial improvements in performance: $J_{\mathrm{sc}}$ increases from 15 to $34\;\mathrm{mA}\;{\mathrm{cm}}^{-2}$, $V_{\mathrm{oc}}$ from 0.54 to above 0.61 V, and the efficiency from 6.5% to 17%. The field and current-flow maps additionally show that the irregular GB network can localize electric fields and redistribute carrier flow. For experiments and processing, the results support both larger grains, which reduce active GB area, and GB passivation, which lowers the recombination velocity of the boundaries that remain.

## Data Availability

The data supporting the findings of this study are available from the corresponding author upon reasonable request.

## Supplementary Materials

The following supporting information can be downloaded at https://www.mdpi.com/article/10.3390/nano16161041/s1, Figure S1: dependence of fill factor on grain-boundary trap density and capture cross-section; Figure S2: total recombination rate versus grain-boundary angle; Figure S3: fixed-length grain-boundary geometry; Figure S4: current–voltage characteristics for five-boundary configurations; Figure S5: local electrostatic potential and current flow around an active grain boundary; Figure S6: electron and hole current flow in an irregular grain-boundary network.

## Abbreviations

The following abbreviations are used in this manuscript:

2D	Two-dimensional.
GB	Grain boundary.
IBC	Interdigitated back contact.
PERC	Passivated emitter and rear contact.
SHJ	Silicon heterojunction.
SRH	Shockley–Read–Hall.
TOPCon	Tunnel oxide passivated contact.
