# Supplementary material for: From Individual Grain Boundaries to Irregular Grain Networks: Drift–Diffusion Simulation of Polycrystalline Silicon Solar Cells
Source: Nanomaterials (Basel). 2026 Aug 21;16(16):1041. doi: 10.3390/nano16161041 (PMC13516571; doi:10.3390/nano16161041)
Supplement: Supplementary file 1 [file nanomaterials-16-01041-s001.zip › nanomaterials-4498200-supplementary.pdf]

Supplementary Materials: From Individual Grain Boundaries to Irregular Grain Networks: Drift–Diffusion Simulation of Polycrystalline Silicon Solar Cells

Irodakhon Gulomova [1,*], Oussama Accouche [2], Zaher Al Barakeh [2], Rayimjon Aliev [1], Navruzbek Mirzaalimov [1], Makhfuza Alinazarova [3] and Jasurbek Gulomov [1,4]

The main article shows that increasing the grain-boundary trap density, N_t, or carrier capture cross section, σ, strengthens recombination and reduces the photovoltaic performance. Figure S1 shows the corresponding fill-factor map. The fill factor decreases as N_t and σ increase, following the same general trend observed for J_{sc}, V_{oc}, and efficiency.

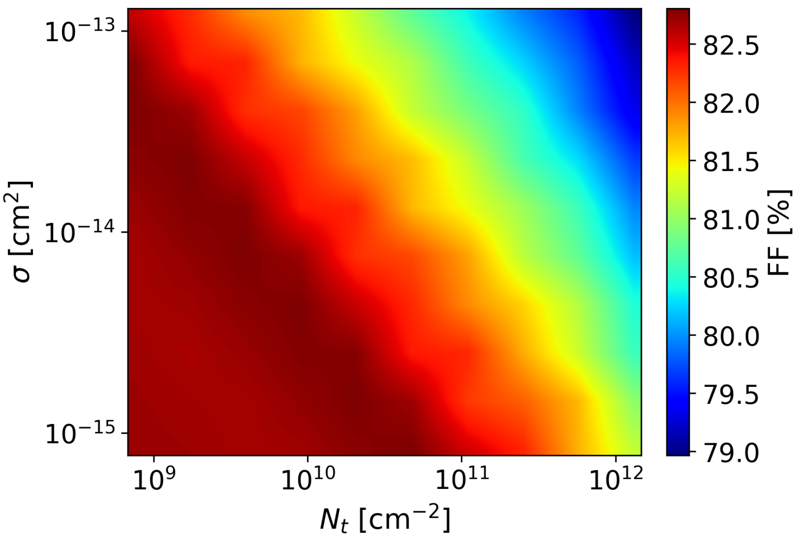

Figure S1. Dependence of the fill factor (FF) on grain-boundary trap density, N_t, and carrier capture cross section, σ, for the silicon solar cell containing a vertical grain boundary.

For the configuration in which the grain boundary remains connected to the device borders, its total length changes during rotation. Figure S2 shows that the total recombination rate increases with grain-boundary angle, reaches a maximum near 75°, and then decreases toward 90°. This behavior follows the strong degradation of the photovoltaic parameters discussed in the main article and supports the conclusion that the changing grain-boundary length is an important contribution to the observed angular dependence.

To separate the effect of orientation from the accompanying change in grain-boundary length, the second configuration keeps the grain-boundary length fixed at 100 μm and rotates it about the center of the device. The corresponding geometry is shown in Fig. S3.

Figure S4 compares the current–voltage characteristics of solar cells containing five vertical, five horizontal, or five randomly arranged grain boundaries. The vertical configuration produces the largest degradation under the present simulation conditions, whereas the horizontal and random configurations show smaller losses. This result further shows that grain-boundary count alone is not sufficient to determine the device performance, because the boundary length and spatial arrangement also play important roles.

To illustrate the local physical processes around a grain boundary, an additional 5×5 μm^2 silicon device was simulated with one highly electrically active grain boundary. The enhanced defect activity was used to make the local electrostatic and transport effects more clearly visible. As shown in Fig. S5, the grain boundary modifies the electrostatic

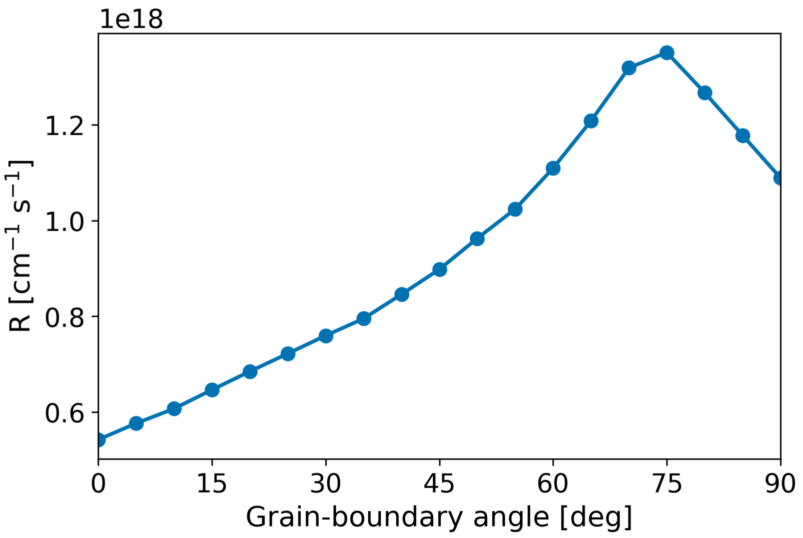

Figure S2. Total recombination rate as a function of grain-boundary angle for the configuration in which the grain-boundary length changes during rotation.

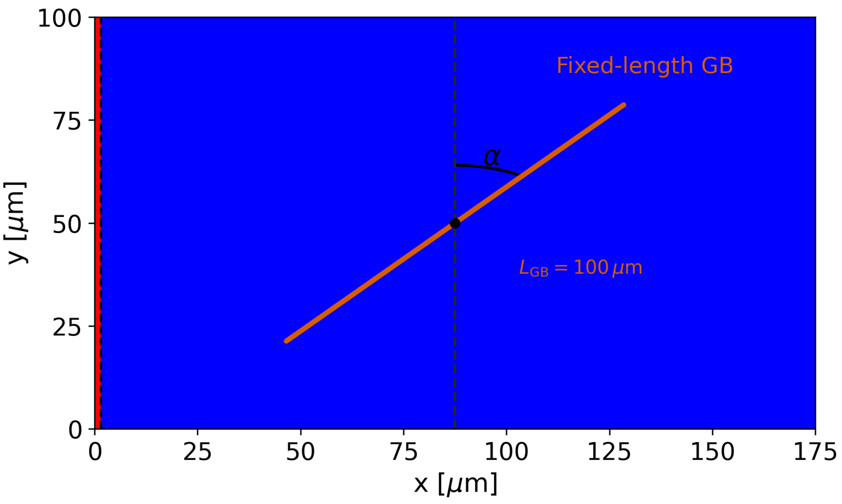

Figure S3. Geometry used for the fixed-length grain-boundary orientation study. The grain boundary has a constant length of $L_{GB} = 100\ \mu$m and is rotated by an angle α about the center of the simulated device.

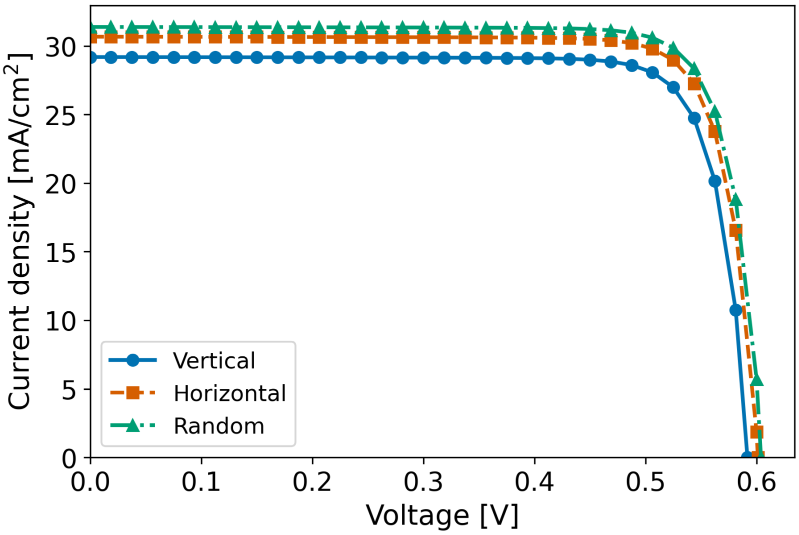

Figure S4. Current–voltage characteristics of silicon solar cells containing five vertical, five horizontal, or five randomly arranged grain boundaries.

potential and redistributes the total, electron, and hole current flow. The current streamlines are strongly distorted near the grain boundary and its intersection with the n/p junction.

The main article presents the electric-field distribution and total current flow for an irregular Voronoi grain-boundary network. Figure S6 shows the corresponding electron and hole current-density distributions separately. The electron current is strongly redistributed near the grain boundaries and their junctions, while the hole-current pathways are also modified by the irregular network geometry.

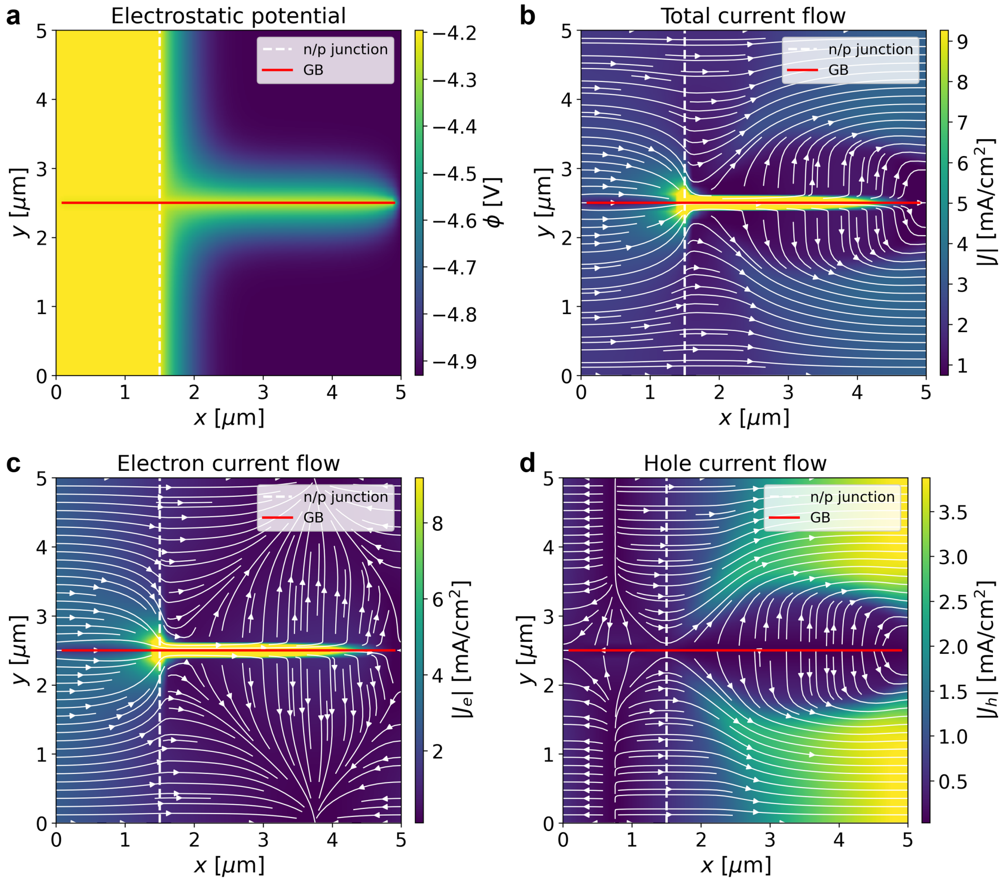

Figure S5. Local electrostatic and current-flow response of a 5×5 μm^2 silicon solar-cell structure containing one highly electrically active grain boundary. **a** Electrostatic potential. **b** Total current-density magnitude and streamlines. **c** Electron current-density magnitude and streamlines. **d** Hole current-density magnitude and streamlines. The dashed white line marks the n/p junction and the red line marks the grain boundary.

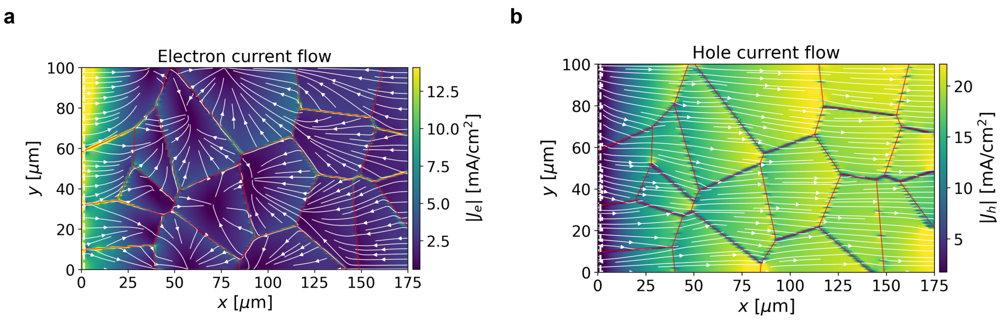

Figure S6. Spatial distributions of carrier current flow in a silicon solar cell containing an irregular Voronoi grain-boundary network. **a** Electron current-density magnitude and streamlines. **b** Hole current-density magnitude and streamlines. Red lines indicate grain boundaries.
